# Supplementary material for: Some like it hot: a differential response to changing temperatures by the malaria vectors Anopheles funestus and An. gambiae s.l
Source: PeerJ. 2017 Mar 28;5:e3099. doi: 10.7717/peerj.3099 (PMC5372839; doi:10.7717/peerj.3099)
Supplement: Data S1 [file peerj-05-3099-s013.docx]

Column headings Supplementary flie 3

Sheet Pessoas – People

1. # casa –House number
2. Nome - Name
3. Data Nasc – Birthdate
4. Sexo –male or female
5. Rede - Do they own a mosquito net?
6. PIN Personal Identity number
7. Cartao – Health post cards distributed
8. Onde foi? What happened to it
9. Observacoes – Observations

Sheet Animais

1. Casa –House number
2. Nenhuma – no animals
3. Cao – dog
4. Porco –pig
5. Boi –cow
6. Gato –cat
7. Pato – duck
8. Cabrito – goat
9. Galinha – chicken
10. Peru – turkey
11. Outros –others

Sheet Casas

1. Casa – house
2. Data –date of survey
3. Entrevistada – name of surveyor
4. D da casa – house owner
5. Tempo aqui – how long have they lived there?
6. Pessoas –number of people in the house
7. Nome de for a –name of ‘resident’ who is away (in mines of South Africa)
8. Mortos –how many people died in the last three years?
9. Potavel – where do they get their drinking water?
10. Lavar – where do they get water /do washing?
11. Animais – are there animals?
12. quartos – How many rooms?
13. Camas – How many beds?
14. Janelas – are there windows?
15. Telhado- material of the roof
16. Aberturas – are there openings at the gable ends that allow ingress to mosquitoes?
17. Porto – Material of the door.
18. Dirrecao – direction that the door faces.
19. Paredes – material of the walls
20. Cumprimento- length in cms
21. Largura –width in centimeters
22. Sabe a venda? –do you know where mosquito nets can be bought?
23. Preco – do you know how much they cost?
24. Doente faz? – when ill where do you go?
25. Conhece o posto? – do you know of the project health post?
26. Do you know what the post treats?
27. Cartas – do you have PIN cards?

AB –novos – new card issued

AC GPS – have co-ordinates been taken?

Sheet – mosquiteiros

1. # casa –house number
2. imprgnado – was the net impregnated at any time?
3. Quando – when?
4. Preco – how much did they pay for their net (in old meticais)

Sheet mortos

1. # casa –house number
2. Nome – name
3. Idade – age
4. Age 1 modified to group
5. Age if exact age unknown
6. Age group
7. Sexo –sex male or female
8. Causau do morte – cause of death
9. Columns J to AB give the cause of death

The column headings for Supplementary files 4 (a garmin database) and 5 (an excel database derived from the delta logger) in supplementary file 5 the sheets with a year label give daily records of the rain measured in Maxixe kindly supplied by RioSul. The other sheets provide the analysis of these data by ISO week. Supplemental file 6 has a sheet that provides the definitions for the column abbreviations – other sheets are self explanatory

Supplementary file 8 (An. gambiae PCR data)

1. Lab – lab where the analysis was conducted
2. N sample number
3. Lot sample group
4. Tube –eppendorf number (for reference)
5. Size wing length of the mosquito

Other columns self explanatory

Supplemental file 9 (identification of An. funestus) is also self explanatory (at least for the relevant columns)

Column headings for Supplementary file 10 RAW data

Sheet RAW data

1. Number –reference number
2. Sheet – number of hard copy sheet for data checking
3. Day
4. Month
5. Year of collection
6. House –House number
7. Collection – collection type
8. Nr copo – cup number
9. Treatment – if any intervention is in place
10. Period – period of the night
11. Start time
12. End time
13. Net type – in use in the house
14. Name – name of collector
15. Af unfed etc until AM

AM Marcado – number of marked An funestus females in the collection (if any)

AN – colour – of the mark

AO – marcadoM – number of male An. funestus marked

Then Ag and Cx. (irrelevant)

Supplementary file 12 has a sheet with the explanation of the column headings
